# A comprehensive approach to the segmentation of multichannel three-dimensional MR brain images in multiple sclerosis^☆^

**DOI:** 10.1016/j.nicl.2012.12.007

**Published:** 2013-01-11

**Authors:** Sushmita Datta, Ponnada A. Narayana

**Affiliations:** Department of Diagnostic and Interventional Imaging, The University of Texas Medical School at Houston, Houston, TX 77030, USA

**Keywords:** Brain, Deformation, Multichannel MRI, Multiple sclerosis, Segmentation

## Abstract

Accurate classification and quantification of brain tissues is important for monitoring disease progression, measurement of atrophy, and correlating magnetic resonance (MR) measures with clinical disability. Classification of MR brain images in the presence of lesions, such as multiple sclerosis (MS), is particularly challenging. Images obtained with lower resolution often suffer from partial volume averaging leading to false classifications. While partial volume averaging can be reduced by acquiring volumetric images at high resolution, image segmentation and quantification can be technically challenging. In this study, we integrated the brain anatomical knowledge with non-parametric and parametric statistical classifiers for automatically classifying tissues and lesions on high resolution multichannel three-dimensional images acquired on 60 MS brains. The results of automatic lesion segmentation were reviewed by the expert. The agreement between results obtained by the automated analysis and the expert was excellent as assessed by the quantitative metrics, low absolute volume difference percent (36.18 ± 34.90), low average symmetric surface distance (1.64 mm ± 1.30 mm), high true positive rate (84.75 ± 12.69), and low false positive rate (34.10 ± 16.00). The segmented results were also in close agreement with the corrected results as assessed by Bland–Altman and regression analyses. Finally, our lesion segmentation was validated using the MS lesion segmentation grand challenge dataset (MICCAI 2008).

## Highlights

► Segmentation of 3D multichannel brain images to various tissues and lesions ► Integration of statistical classifiers and brain anatomy improves segmentation. ► Automated technique minimizes user intervention with reproducible results. ► Quantitative evaluation shows excellent tissue and lesion classification.

## 1 Introduction

Magnetic resonance imaging (MRI) plays a major role in quantifying brain lesions and other tissues for assessing the disease course, understanding the underlying pathophysiology, and investigating the therapeutic efficacy in multiple sclerosis (MS). Image segmentation or tissue classification is one of the critical steps for MR image analysis. On MRI, T2 hyperintense white matter (WM) (THWLs), T1 hypointense (or black holes), Gd-enhanced, and cortical lesions are seen. Both lesion volumes and/or number and their locations appear to influence the clinical disability, including cognitive impairment (Bodini et al., 2011; Fisniku et al., 2008; Kincses et al., 2011; Mostert et al., 2010; Patti et al., 2009; Poonawalla et al., 2010; Rudick et al., 2006; Sastre-Garriga and Tintore, 2010; Tao et al., 2009a; Vellinga et al., 2009).

Automated or minimally operator dependent segmentation of gray matter (GM), WM, cerebrospinal fluid (CSF) and lesions is desirable to reduce operator bias and for reproducible results. Classification of MS lesions is challenging due to their diffuse nature along the boundary and large intensity variations. Improper tissue classification could affect measures such as GM and WM atrophy which appear to correlate with clinical disability (Chard et al., 2010; Derakhshan et al., 2010; Grassiot et al., 2009).

Several semi-automated and automated techniques have been developed for classifying lesions using images acquired in two-dimensional (2D) or three-dimensional (3D) mode with single or multiple MRI sequence(s). The multiple sequences include proton density-weighted (or PD), T1-weighted (or T1), T2-weighted (or T2), and Fluid Attenuated Inversion Recovery (FLAIR). Kamber et al. (1995) developed geometric brain tissue probability maps of extracortical CSF, ventricular CSF, GM, and WM to classify lesions based on the 2D dual echo (PD & T2) images with the decision tree classifiers. Akselrod-Ballin et al. (2009) applied a decision tree classifier based on intensity, shape, location, neighborhood relationships, and anatomical context to classify lesions on the multichannel 2D (PD, T1, & T2), and 3D FLAIR images. Udupa et al. (1997) proposed a semi-automated classification technique based on the 2D dual echo (PD & T2) images where a number of training points for GM, WM, and CSF were selected by the operator and identified based on the fuzzy-connectedness principle, leaving holes within these structures to be considered as possible lesions. Horsfield et al. (2007) further modified this technique to reduce operator's time in the final identification of lesions by including information about the spatial distribution and size characteristics of MS lesions. The modified technique was applied to 2D dual echo (PD & T2) images.

A parametric approach, expectation-maximization (EM), was proposed by Wells et al. (1996) for iterative classification of tissues along with bias correction and was implemented on the 2D axial dual echo and 3D T1 images of a normal brain. The contextual information was included using hidden Markov random field (HMRF) (Zhang et al., 2001) for improved classification. These techniques assume that tissues follow the Gaussian distribution. To improve the lesion classification in MS, van Leemput et al. (2001) assumed lesions to be outliers and applied the same approach to the multichannel 2D (PD, T1, & T2) images.

Non-parametric techniques do not assume any distribution for the tissues and the classification is based on the feature maps generated with feature vectors containing the training points selected from each tissue and lesions. Jackson et al. (1993) have applied the Parzen classifier for identifying lesions along with CSF and brain regions on the 2D dual echo images. Anbeek et al. (2004) applied the k-nearest neighbor (k-NN) classifier to segment the WM lesions using information from the multichannel 2D (PD, T1, T2, IR, & FLAIR) images. Sajja et al. (2006) combined the parametric and non-parametric techniques for segmentation of tissues and lesions based on the multichannel 2D (PD, T2, & FLAIR) images. False lesion classifications were minimized using the ratio of PD and T2 images.

Zijdenbos et al. (2002) used supervised artificial neural network technique using image intensity and spatial priors to classify lesions on the multichannel (3D T1, and 2D PD & T2) images. Lao et al. (2008) proposed a computer-assisted technique for classifying lesions on the multichannel 2D (PD, T1, T2 & FLAIR) images using a set of training samples manually delineated by experts and support vector machine (SVM) based classification. Shiee et al. (2010) proposed a segmentation technique based on the topological and statistical atlases to classify tissues/lesions on the simulated multichannel 2D (PD, T1, & T2) and real T1 and FLAIR images. Garcia-Lorenzo et al. (2011) implemented a trimmed likelihood estimator initialized with a hierarchical random approach to classify the lesions along with the brain tissues using the 2D (PD & T2), and 3D T1 images by co-aligning the T1 to T2 images. Geremia et al. (2011) used a spatial decision forest classifier to segment lesions on the multichannel 2D (T1, T2, & FLAIR) images.

Wu et al. (2006) combined the intensity-based statistical k-NN classification with template-driven segmentation and partial volume averaging correction for classifying the MS lesion subtypes (T2 hyperintense, T1 hypointense, and Gd-enhanced lesions) along with other tissues on the multichannel 2D (PD, T1 & T2) images using operator-supervised tissue sampling and parameter calibration. Datta et al. (2006, 2007) proposed automated segmentation techniques based on morphological grayscale reconstruction techniques for classifying T1 hypointense and Gd-enhanced lesions using the multichannel 2D (PD, T1, T2 & FLAIR) images.

T2 hyperintense WM lesions are also present in elderly brains or brains affected by other neurological diseases such as Alzheimer's disease. Admiraal-Behloul et al. (2005) utilized an artificial intelligence technique to classify these lesions by combining information from the multichannel 2D (T2, PD, & FLAIR) images. Ramirez et al. (2011) proposed a lesion explorer technique for identifying the subcortical hyperintensities in Alzheimer's disease. These authors adapted a local thresholding model to identify and isolate the subcortical hyperintensities from the periventricular hyperintensities using the 3D T1 and 2D dual echo images. The same approach could be adapted for segmenting MS brains. However, this technique is based on a local threshold that can vary from subject to subject, making it difficult to apply consistently.

The majority of the above lesion segmentation techniques are based on the 2D images. In general, the 2D images have limited spatial resolution, especially along the slice direction. In particular, the spatial resolution affects the quantitative analysis of tissues and lesions due to partial volume averaging effect. Segmentation based on the 2D images may be sub-optimal in some regions, particularly in the cerebellum and vertex regions, and the deep GM (or dGM) structures (Datta et al., 2009, 2011a; Derakhshan et al., 2010).

Segmentation based on the multichannel 3D images can overcome many of the problems indicated above. With the introduction of parallel imaging along with the general availability of high field MRI scanners, high resolution three-dimensional (3D) images can be acquired in a clinically acceptable time. There is a general agreement that 3D imaging would be routinely performed in patient management and clinical trials in the near future (Barkhof et al., 2011). It was also shown that 3D imaging of MS brain acquired on 3 T scanners provides better identification of lesions compared to 1.5 T (Bagnato et al., 2006; Barkhof et al., 2011; Dolezal et al., 2007; Nelson et al., 2008; Sicotte et al., 2003; Simon et al., 2010). In addition to the higher sensitivity in detecting lesions, 3D images provide high signal-to-noise ratio (SNR) and are less affected by flow artifacts compared to the 2D images. Flow artifacts in the 2D images often lead to false lesion classifications. An additional advantage of the 3D images with isotropic resolution is that images can be viewed in any plane without the interpolation artifacts. Although 3D images have higher SNR compared to 2D images, they also suffer from high intensity non-uniformity and larger data volumes and the existing semi-automated or manual processing techniques are not well suited.

In the present study, we developed and implemented a comprehensive and automated segmentation technique that combines non-parametric and parametric statistical techniques to classify tissues/lesions on the high resolution 3D images. The proposed technique for the 3D analysis is based on our previous technique developed for the 2D analysis (Sajja et al., 2006) and includes novel features to improve the overall classification while automating the technique. These novel features include: (1) automated brain extraction using the T2 images with fat-sat technique, (2) isolating and segmenting the cerebellum for superior GM–WM classification, and (3) integrating anatomical knowledge using the brain template for minimizing false classifications and improving the parcellation of GM structures. In this study, the focus is only on the classification of THWLs along with GM, WM, and CSF. We also validated the THWL classification using quantitative analyses. In addition, the technique was validated on the private data using the MS lesion segmentation grand challenge dataset (MICCAI 2008). In the remainder of the manuscript, lesions refer to THWLs.

## 2 Materials and methods

### 2.1 Subjects and MRI-protocol

Sixty (46 females and 14 males) patients with clinically definite relapsing remitting MS (RRMS) with a median age of 47.5 years (range: 18–66 years) were included in this study. Written informed consent was obtained from all the subjects. These studies were approved by our Institutional Committee for the Protection of Human Subjects and are HIPAA compliant.

A 3 T Philips Intera scanner with a quasar gradient system (Philips Medical Systems, Best, Netherlands) capable of producing maximum gradient amplitude of 80 mT/m (slew rate 200 mT/m/ms) and an eight channel head coil was used to acquire the whole brain MR images. Three-dimensional magnetization prepared rapid gradient echo (MPRAGE) based T1 and turbo-spin echo based T2 and FLAIR images with 1 mm^3^ isotropic voxel resolution were acquired in the sagittal plane. The sequence parameters and the acquisition times are summarized in Table 1. The T2 images were acquired using the fat saturation (fat-sat) technique for suppressing fatty tissues between the brain and the skull for automated skull stripping (Datta and Narayana, 2011). Due to long acquisition time, the 3D PD images were not acquired.

Additional data was obtained from the MS lesion segmentation grand challenge (Medical Image Computing and Computer Assisted Intervention, MICCAI 2008) that provides MS brain images for validating lesion classification technique (http://www.ia.unc.edu/MSseg/). There are two datasets, referred to as the public and private data. There are 10 scans each in the University of North Carolina (UNC) and the Children's Hospital in Boston (CHB) cohorts in the public data and also includes expert lesion classification. The private data includes 13 scans from the UNC and 17 scans from the CHB cohorts, respectively. Lesion classification on the private data obtained by the CHB and the UNC experts are not available to the public. All the scans include T1, T2 and FLAIR images that are co-registered and re-sampled to 512 × 512 × 512 with voxel dimensions of 0.5 × 0.5 × 0.5 mm^3^ (Styner et al., 2008).

### 2.2 Methods

**Fig. 1:**
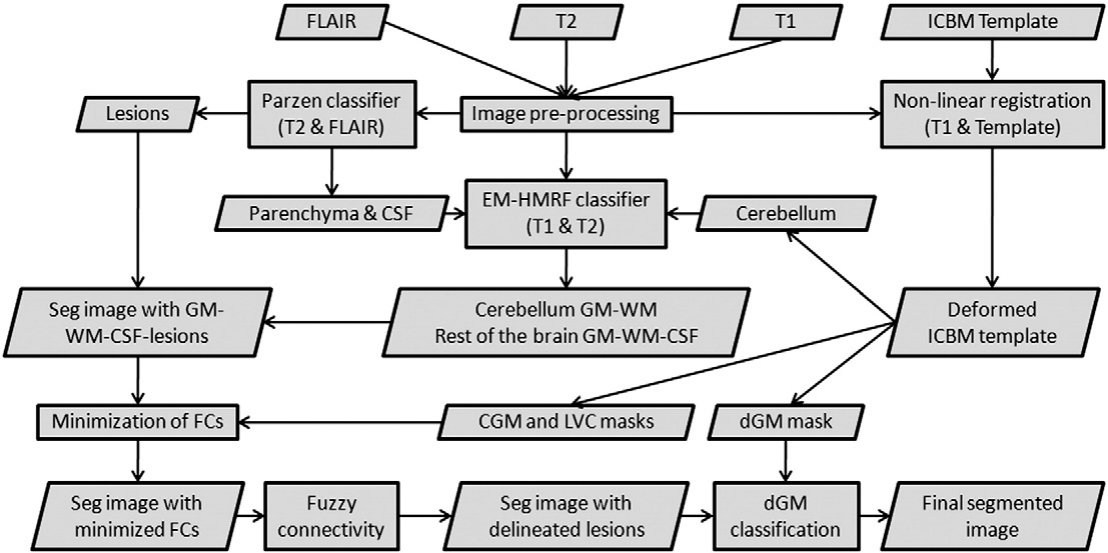
Schematic diagram summarizing the steps involved in the automated segmentation of tissues and lesions in MS. Image modalities used at each step are included in parentheses. Here, FCs stands for false classifications.

The four major components of the proposed segmentation technique are: (1) image pre-processing, (2) tissue/lesion classification, (3) minimization of false lesion classifications, and (4) classification of dGM structures (Datta et al., 2011b). A flow chart with an overview of the proposed method, including the use of multichannel MRI in each step, is presented in Fig. 1. Lesion segmentation obtained on the data from 3 T Philips scanner was qualitatively analyzed and corrected for false classifications by the expert, who has 20 + years of experience in MRI, MS, and neuroanatomy. The sagittal images acquired with an isotropic resolution were reformatted into axial format prior to processing.

### 2.3 Image pre-processing

The image pre-processing includes registration for aligning all images into the same coordinate system, brain extraction, intensity non-uniformity correction, and noise reduction. Although the images were acquired during the same session, a certain amount of subject motion and movement is unavoidable between the sequences, leading to image misalignment. For each subject, the T1 and FLAIR images were aligned with the T2 images using a 3D rigid body image registration algorithm proposed by Ashburner et al. (2000) (SPM2; http://www.fil.ion.ucl.ac.uk/spm/software/spm2/). Since all the images were acquired in the same session, application of rigid body registration is adequate.

The majority of brain extraction techniques usually focus on the T1 images (Derakhshan et al., 2010; Garcia-Lorenzo et al., 2011; Lao et al., 2008; Shiee et al., 2010). Brain extraction from the T1 images often results in the loss of extracortical (sulcal and subarachnoid) CSF leading to the underestimation of CSF volume (Ramirez et al., 2011). To overcome this problem, we have applied automated brain extraction algorithm to the T2 images to remove extrameningeal tissues by exploiting the fat-sat technique and applying image histogram-based thresholds as described elsewhere (Datta and Narayana, 2011). This skull stripped T2 image was used to generate the mask which was applied to the co-aligned T1 and FLAIR images.

The skull-stripped images were corrected for intensity nonuniformity using the module in SPM2 (SPM2; http://www.fil.ion.ucl.ac.uk/spm/software/spm2/) (Ashburner, 2002) followed by the application of anisotropic diffusion filter to reduce noise while preserving the edges (Gerig et al., 1992; Perona and Malik, 1990). Since the segmentation is based on image intensity (see below), the intensity profiles of the images were standardized (or normalized) using the decile based piece-wise linear transformation technique (Nyul et al., 2000). The rigid registration and intensity nonuniformity correction modules in SPM2 that were used in preprocessing were rewritten in IDL and integrated into our segmentation pipeline. We used the inhomogeneity and rigid body registration modules from the SPM2 since they were integrated as a part of the segmentation pipeline in SPM5 and SPM8.

### 2.4 Tissue/lesion classification

The intracranial brain was classified into GM, WM, CSF, and lesions by combining parametric and non-parametric techniques with the integration of brain anatomical knowledge (see below). In the first step, the parenchyma, CSF, and lesions were classified using the non-parametric technique, the Parzen window classifier. Initially, the expert with 20 + years of experience in MRI, MS, and neuroanatomy identified voxels that represent GM, WM, CSF, and lesions on the T2 and FLAIR images. These are referred to as the training points. The numbers of training points selected for GM, WM, CSF, and lesions were 100, 111, 124, and 168, respectively. These training points were selected from 14 randomly chosen T2 and FLAIR images. A two-dimensional feature map was generated using the Gaussian kernel on the training points (Duda et al., 2001). Because the images were intensity normalized as a part of the image preprocessing, the feature map needed to be generated only once. This feature map was applied to all the images for Parzen classification (Sajja et al., 2006). The feature map was applied to the T2 and FLAIR images to classify tissues into parenchyma, CSF, and lesions. Only lesions obtained from the Parzen classification were retained as the lesion class. Since the lesions do not follow the Gaussian distribution, the lesion voxels were removed from the pre-processed images following the Parzen classification. The remaining brain voxels were classified into GM, WM, and CSF as described below.

Although it is widely assumed that the intensity profile of a given tissue (other than lesions) is the same throughout the brain, independent of the spatial location, the intensity profiles of GM and WM in the cerebellum generally differ from the rest of the brain (Datta et al., 2009; Xiao et al., 2010). To address the effect of this intensity variation on tissue classification, the cerebellum was automatically isolated from the rest of the brain and these regions were segmented independently, as described by Datta et al. (2009). Briefly, the ICBM (International Consortium for Brain Mapping) template (http://www.loni.ucla.edu/ICBM/Downloads/Downloads_ICBMtemplate.shtml) was skull-stripped with the Brain Extraction Tool (BET) (Smith, 2002) and co-aligned to the subject's skull-stripped T1 image using symmetric diffeomorphic non-linear registration (Avants et al., 2008; Datta et al., 2009). The template was produced by averaging 27 T1-weighted MRI acquisitions from a single subject (Montreal Neurological Institute database) and was parcellated into 55 GM structures that include the cerebellum and WM (single structure). The deformation field obtained from the non-linear registration was applied to deform the ICBM template to isolate the cerebellum from the brain on subject's T1 images. The cerebellum was segmented into GM and WM, whereas the remaining part of the brain was classified into GM, WM, and CSF using the parametric technique, expectation-maximization with hidden Markov-random field (EM-HMRF) algorithm (Zhang et al., 2001). Assuming that the tissues follow a multivariate Gaussian distribution, this algorithm classifies the tissues iteratively with bias correction while incorporating the contextual constraints on the pair of skull-stripped T1 and T2 images. Recent studies showed that tissue segmentation based on multi-channel images yields superior results relative to the segmentation based on T1-weighted images alone (Bazin and Pham, 2008; Derakhshan et al., 2010; Wels et al., 2011). The initial inputs required for the EM-HMRF algorithm for the classification of GM and WM were obtained from the parenchyma class with the application of the Parzen classifier on the T1 and T2 images (Sajja et al., 2006). The CSF class, obtained from the T2 and FLAIR images, was used as the input for CSF classification. Following the final classification into GM, WM, and CSF on the T1 and T2 images, the classified tissues were combined with the lesion class, obtained earlier with the Parzen classifier, for whole brain segmentation.

### 2.5 Minimization of false lesion classifications

With any segmentation technique certain false classifications are unavoidable. These false classifications often occur with our procedure since not all hyperintense voxels represent lesions on the T2 and FLAIR images. As described below, the anatomical information on the previously co-aligned ICBM template was used to minimize these false lesion classifications.

#### 2.5.1 Hyperintense voxels not associated with lesions

The non-lesion hyperintense voxels were also classified as lesions by the Parzen classifier. To minimize these false classifications, we assumed that lesions are not present in the cortex and/or at the GM/CSF boundary. This assumption is justified since the focus of this work is on the classification of tissues and WM lesions only. In addition, cortical lesions tend to be small and are infrequently seen on the FLAIR and T2 images (Nelson et al., 2008). For minimizing the false lesion classifications, the cortical GM (CGM) mask from the previously co-aligned ICBM template was obtained (Nakamura and Fisher, 2009). Any regions appearing hyperintense on both the T2 and FLAIR images and classified as lesion, but were within the CGM mask, were removed from the lesion classification and re-classified as GM. This procedure also removes cortical lesions, if any, identified on the FLAIR and T2 images. A very small subset of false classification is also seen within 2 to 3 voxels from the brain surface. They arise from the misalignment (which occurs very rarely) of the FLAIR with T2 images following the application of rigid body registration. This subset of false classifications was removed by the application of morphological erosion technique (Sajja et al., 2006). The false lesion classifications were further minimized by reclassifying the lesions comprising of 3 voxels or less as GM. Due to their diffuse nature (particularly at the edges), the size of the lesions is often underestimated and therefore a fuzzy connected algorithm was applied to properly delineate the lesions (Udupa et al., 1997). The two parameters, fuzzy adjacency and fuzzy affinity, associated with the fuzzy connectivity technique were optimized as described elsewhere (Datta et al., 2006). The optimized parameters were applied across all the images following intensity standardization.

#### 2.5.2 Choroid plexus misclassified as lesions

Choroid plexus within the lateral ventricular CSF (LVC) appears hyperintense on both T2 and FLAIR images and is generally classified as lesions. Elimination of these false positives requires generation of the LVC mask. First, the LVC was automatically identified on the deformed anatomical image volume. Morphological dilation and closing operations were applied to smooth out the boundaries, eliminate any voxels with partial volume averaging, and fill up any holes that might have appeared due to the deformation procedure (Ramirez et al., 2011). Using this LVC mask, the choroid plexus, classified as lesions on the segmented images, was re-classified as CSF.

### 2.6 Classification of the dGM structures

Unlike CGM, the dGM structures in MS are difficult to segment accurately as they tend to appear hypointense on the T2 images (Bakshi et al., 2002). In the present study, we observed that the EM-HMRF algorithm underestimated the dGM structures. Derakhshan et al. (2010) have also demonstrated the underestimation of GM within these structures. As reported by Tao et al. (2009b), these structures can be segmented accurately by using the deformation field obtained from the non-linear symmetric diffeomorphic registration technique. Therefore, we used the previously co-aligned ICBM template with the subject T1 image to identify the dGM structures. These structures were finally included in the GM class. In this study, this procedure was implemented to re-classify major dGM structures such as thalamus, putamen and caudate nucleus. These structures were partially classified as WM by EM-HMRF.

### 2.7 Lesion classification evaluation

In the absence of ground truth, lesions corrected by the expert were considered as the reference lesion classification. Given the complexity of cerebral anatomy, manual segmentation on a large number of images is tedious, unreliable, and unrealistic (Derakhshan et al., 2010). Therefore, quantitative analysis was performed only on the lesions. The correction of lesion classification was performed by the expert with 20 + years of experience in MRI, MS, and neuroanatomy using the in-house developed validation software (Datta et al., 2006; Sajja et al., 2006) by manually editing out both false positive and negative lesion classifications on the segmented images. This custom and user-friendly software incorporates various tools such as eraser, paint brush, zoom, intensity window and level, and the 3D image viewer to display images simultaneously in axial, sagittal, and coronal planes. Due to the large data size (60 subjects and 3 image volumes, each containing > 150 sections) and the diffuse nature of lesions, it is extremely cumbersome for any expert to manually delineate the lesions based on the raw images. Therefore, the expert relied on the automated lesion classification as the starting point. Validation of lesions was performed based on signal intensity, morphology (including size and shape), location, regional spatial relation, and anatomical context. Signal intensity was evaluated using the T2, FLAIR, and T1 images. Images were visualized in the three orthogonal planes (axial, coronal, and sagittal) simultaneously with the 3D image viewer to establish anatomical location and lesion extension. The hyperintense regions comprising of more than 3 voxels on the FLAIR images with corresponding hyperintensities on the T2 images were regarded as possible lesions. Occasionally, the T1 hypointensities provided much needed indication for identifying those lesions that appeared hyperintense on FLAIR, hypointense on the T1 and subtle hyperintense on the T2 images. The anatomical context was exploited to exclude blood vessels, choroid plexus, flow artifacts or other normal tissues as a source of hyperintensity. True positives were selected as lesions if the algorithm identified them as lesions, whereas false positives were deleted from the classification and re-classified as suitable tissue class. False negatives were painted using the paint brush to mark them as lesions. The edited set of lesions obtained by the expert was considered as the reference classification for evaluating the proposed segmentation technique. The segmentation results of GM, WM, and CSF were only visually judged by the expert.

Finally, we compared our method with that proposed by Sajja et al. (2006) that was based on 2D images.

### 2.8 Quantitative evaluation

The classified lesions obtained with the automated technique were quantitatively compared with the results obtained by the expert, considered as the reference classification. The quantitative metrics used for the evaluation included absolute volume difference percent (vol diff), average symmetric surface distance (avg dist), true positive rate (TPR), and false positive rate (FPR) defined as:

(1) $$\mathrm{vol}\;\mathrm{diff}=\left(\mathrm{abs}\left(\mathrm{reference}\;\mathrm{volume}–\mathrm{segmented}\;\mathrm{volume}\right)/\mathrm{reference}\;\mathrm{volume}\right)*100.0$$

(2) $$\mathrm{avg}\;\mathrm{dist}=\left[\sum_{x\in \partial \left(\mathrm{Seg}\right)}\min_{y\in \partial \left(\mathrm{Ref}\right)}d\left(x,y\right)+\sum_{x\in \partial \left(\mathrm{Ref}\right)}\min_{y\in \partial \left(\mathrm{Seg}\right)}d\left(x,y\right)\right]/\left[\mathrm{card}\left(\mathrm{Seg}\right)+\mathrm{card}\left(\mathrm{Ref}\right)\right]$$

(3) $$\mathrm{TPR}=\left(\mathrm{TP}*100.0\right)/\left(\mathrm{TP}+\mathrm{FN}\right)$$

(4) $$\mathrm{FPR}=\left(\mathrm{FP}*100.0\right)/\left(\mathrm{FP}+\mathrm{TN}\right)\text{.}$$

Here, ∂(Ref) and ∂(Seg) represent sets of voxels representing the boundaries of the ground truth (Ref) and segmented lesions (Seg). The term card(Seg) denotes the number of boundary voxels of the segmented lesions. The average distance was measured in mm.

### 2.9 Statistical analysis

Bland–Altman analysis was performed to assess the agreement and possible bias between the segmented and corrected results. Bland–Altman technique is a statistical tool often used to assess the bias between two techniques. In this method the difference between the results obtained by two different techniques (bias) is plotted against the average of the results, considered to be the truth (Bland and Altman, 1995). We have plotted the difference in the lesion volumes obtained by the automated technique and the expert (bias) against the average (considered to be the truth) of these lesion volumes.

Correlation analysis was performed to assess the agreement between the lesion volumes obtained by our automated technique and the expert using the Pearson correlation coefficient.

### 2.10 Validation

We validated our lesion segmentation results on the private dataset available from the MS lesion segmentation grand challenge (MICCAI 2008). The dataset was created for MICCAI 2008 conference and can be downloaded for lesion segmentation analysis. The results section on the website displays the results from the MICCAI 2008 contest along with the results posted by other research groups (http://www.ia.unc.edu/MSseg/results_table.php). The scoring on the MICCAI data is derived from vol diff, avg dist, TPR, and FPR. A single expert from CHB and two experts from UNC jointly segmented the lesions on the private dataset. Therefore two sets of expert segmentations were used as the reference for comparing the segmentation on each of the scans (Styner et al., 2008). A score of 90 is considered as equivalent to the classification obtained by the human rater.

To validate the proposed technique, the same feature map and pre-set parameters used for the 60 scans used in our study were applied to the MICCAI data.

## 3 Results

**Fig. 2:**
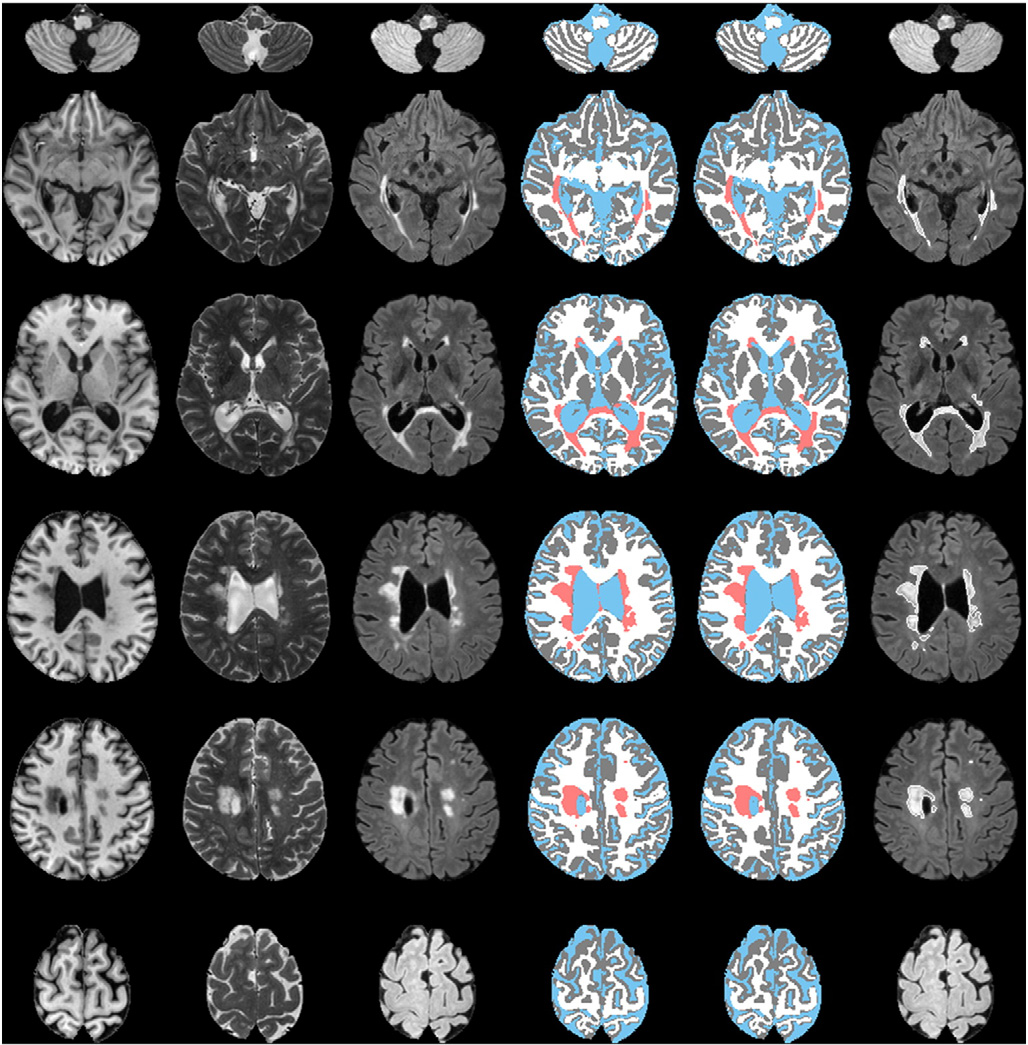
Automated segmentation at various cross-sections from one subject. First column: T1; second column: T2; third column: FLAIR; fourth column: segmented; fifth column: images with corrected lesions; and sixth column: boundaries of corrected lesions superimposed on FLAIR images. GM, WM, CSF, and lesions are represented by gray, white, blue, and salmon colors.

The classification of GM, WM, CSF, and lesions at various cross-sectional levels for one of the MS brains is shown in Fig. 2. The lesions corrected (or edited) by the expert are also included in this figure for reference. The superimposition of corrected lesion boundaries shown on the FLAIR images (sixth column) provides a better visualization of the lesions. Visually, all the tissues including the lesions, especially the GM and WM, were classified very well in the cerebellum and vertex regions, two of the most difficult regions to segment. Consistent results were obtained for all the MS brain images included in this study.

**Fig. 3:**
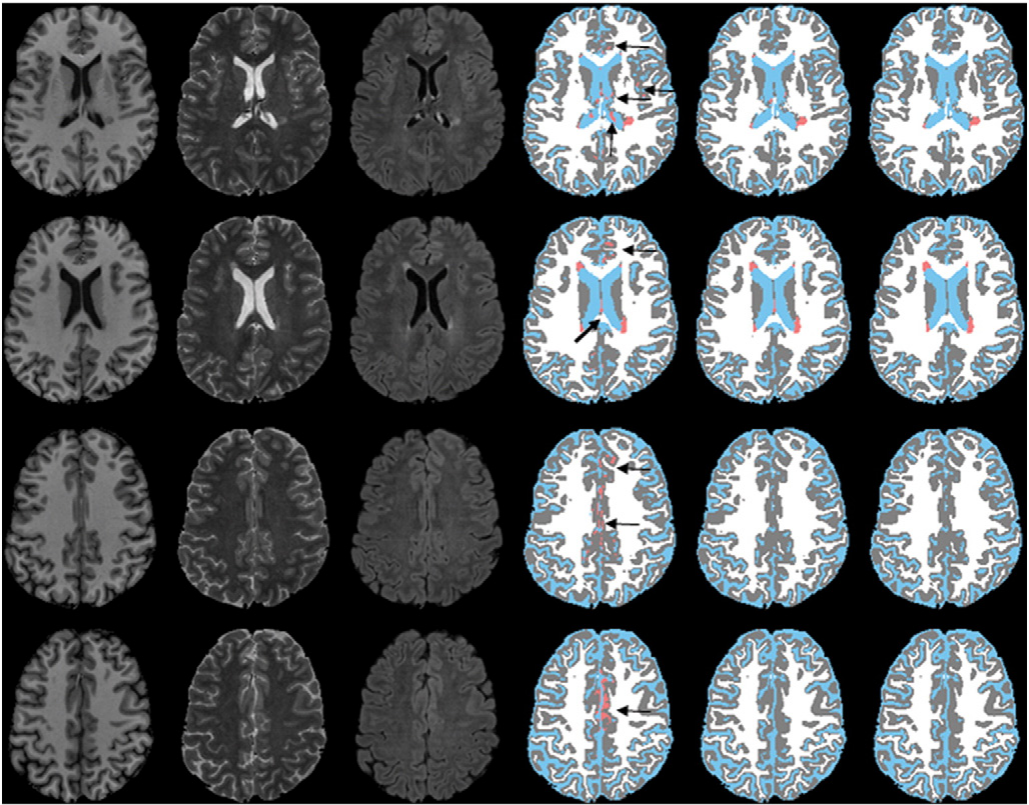
Results of various steps for minimizing the false classifications at different cross-sections from one subject. First column: T1; second column: T2; third column: FLAIR; fourth column: images obtained with the Parzen classification on which false classifications are pointed by arrows; fifth column: images following the minimization of false classifications; and sixth column: images with corrected lesions. Color scheme is same as Fig. 2.

**Fig. 4:**
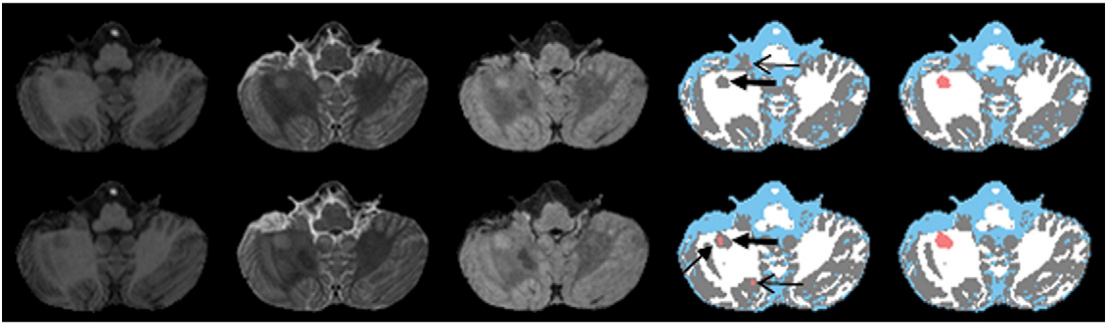
An example of segmentation in consecutive cross-sections of the cerebellum. Color scheme is same as Fig. 2.

An example of the effectiveness of false classification minimization technique is demonstrated in Fig. 3. The arrows in this figure (fourth row) indicate the false classifications following the application of the Parzen and EM-HMRF classifications. Minimization of the false classifications as proposed using the CGM and LVC masks obtained from deformed anatomical images significantly reduced the overestimation of lesions (fifth column) with fewer remaining false classification as shown by the bold arrow. The corrected images were also included in this figure for reference in which the remaining false classifications were removed by the expert. Fig. 4 shows an example of tissue/lesion classification in the cerebellum. Corrected images included in the figure indicate true positive (arrow), false positive (open arrows), and false negative (bold arrows) lesion classifications obtained with the segmentation technique.

**Fig. 5:**
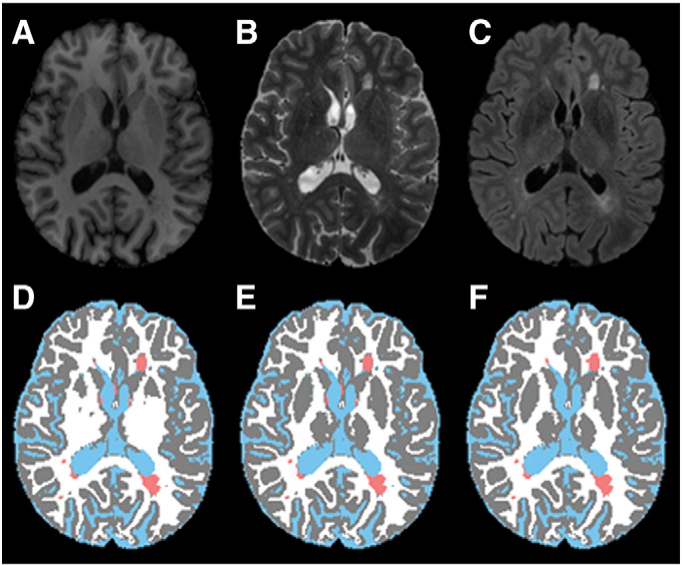
Re-classification of dGM structures. A: T1; B T2; C: FLAIR; D: segmented images; E: following the re-classification of dGM structures using deformed template and its anatomical image; and F: image with corrected lesions. Color scheme is same as Fig. 2.

Fig. 5 shows an example of the dGM segmentation in an MS brain. The EM-HMRF algorithm classified the dGM structures as WM (Fig. 5D). Improvement in the dGM classification following the application of the dGM mask obtained from a deformed anatomical image can be observed in Fig. 5E. With the application of this procedure, the dGM structures were more accurately re-classified as GM in all the MS subjects.

**Fig. 6:**
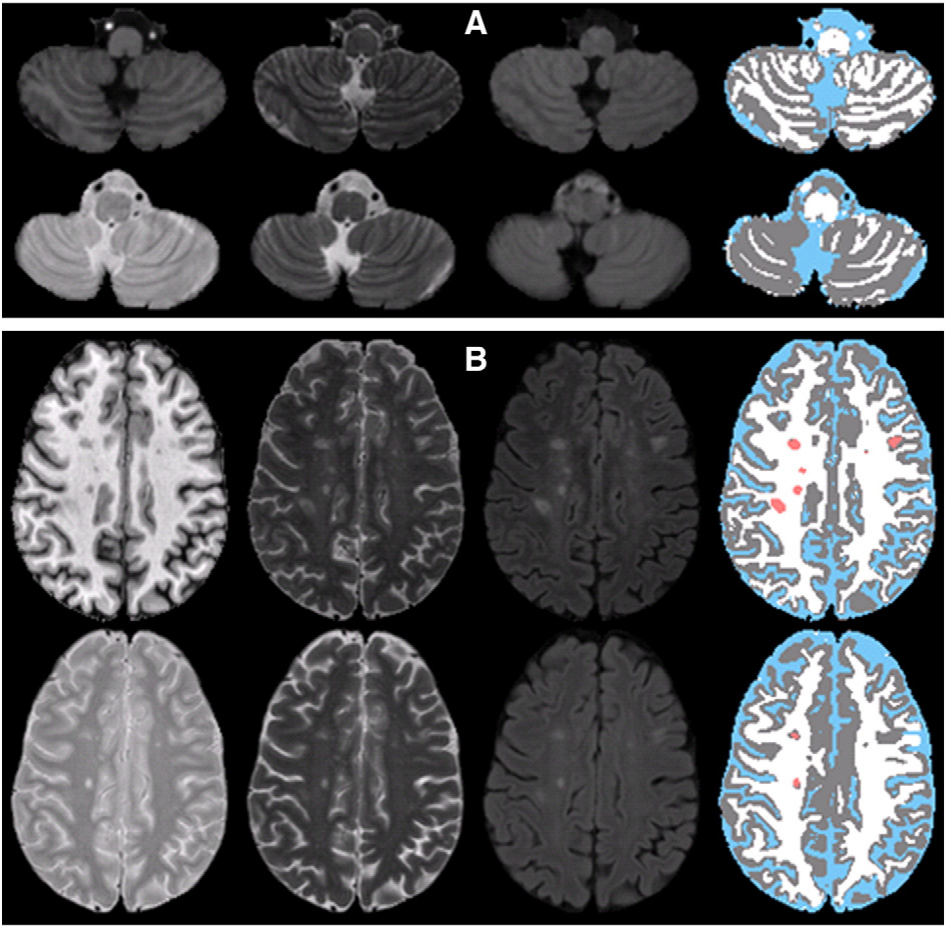
Demonstration of segmentation using the proposed technique on 3D images (T1, T2, and FLAIR) (top row) and using the technique proposed by Sajja et al. (2006) on 2D images (PD, T2, and FLAIR) (bottom row): (A) a section of the cerebellum and (B) the semiovale region. Color scheme is same as Fig. 2.

Fig. 6 demonstrates the comparison of segmentation results obtained on 3D images using our technique with those obtained using the technique proposed by Sajja et al. (2006) on the 2D images at two locations. As can be seen from this figure, the cerebellum in Fig. 6A shows superior GM–WM classification. In addition, lesions are well delineated in Fig. 6B using the proposed technique.

### 3.1 Quantitative evaluation

**Fig. 7:**
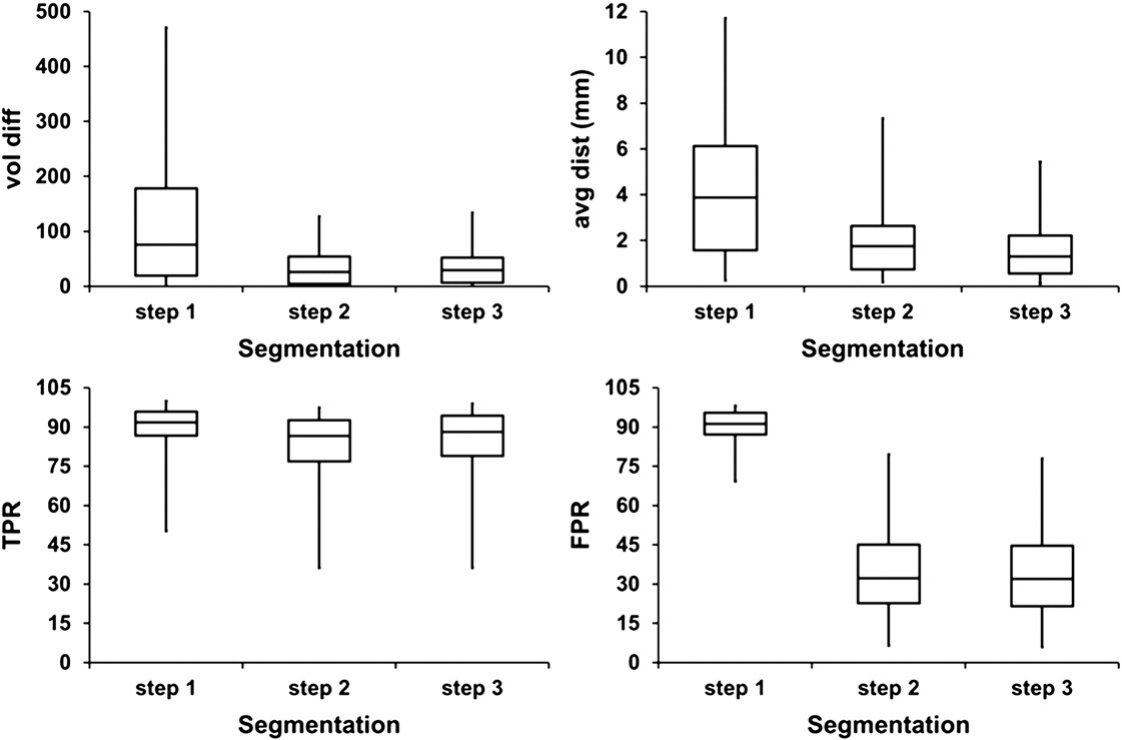
Box-whisker plots of the quantitative measures, vol diff, avg dist, TPR, and FPR for lesions when estimated volumes obtained with the proposed automated technique are compared with volumes corrected by the expert. Here, segmentation steps include application of the Parzen classifier (step 1), minimization of false classifications (step 2) and final segmentation (step 3).

Fig. 7 shows the box-whisker plots of the quantitative metrics, vol diff, avg dist, TPR, and FPR for lesions in all the 60 MS subjects. Box-whisker plot is an appropriate way to represent any set of observation with five variables: minimum, first quartile, median, third quartile, and maximum. This figure clearly demonstrates improvement in the quantitative metrics following the application of each major step in the lesion classification. These major steps include initial classification of lesions, minimization of false classifications, and the application of fuzzy connectivity. As can be seen from Fig. 7, in some instances, the Parzen classifier overestimated the lesion volume by more than 400% as assessed by vol diff. Following the integration of anatomical information about the CGM and LVC, and assuming that the lesions lie within WM, the overestimation was reduced to around 100% of the lesion volume. All the metrics improved following the minimization of false classifications. The application of fuzzy connectivity has considerably improved the avg dist. Overall, significant improvement was observed in the quantitative metrics with the application of each step involved in the classification of lesions.

We also investigated the effect of total lesion volume on the quantitative metrics. The whole database was divided into two groups: group A consists of subjects (31) with lesion volume less than 10 cm^3^ and group B (29) with lesion volume greater than 10 cm^3^. Tables 2–4 provide detailed quantitative metrics, vol diff, avg dist, TPR, and FPR for these two groups prior to and following the false classification minimization and final segmentation (application of fuzzy connectivity) steps. These tables suggest that the lesion volume was consistently overestimated in subjects with lower lesion volumes compared to those with higher lesion volumes. Also, the average symmetric surface distance in group B is higher than that in group A indicating the slightly poor performance of the proposed technique in subjects with low lesion volumes.

### 3.2 Statistical analysis

**Fig. 8:**
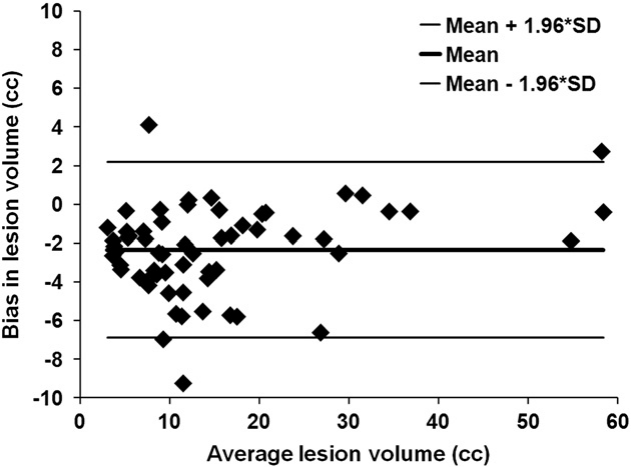
Bland–Altman plot demonstrating the agreement and bias in estimating the lesion volumes with the automated segmented technique.

**Fig. 9:**
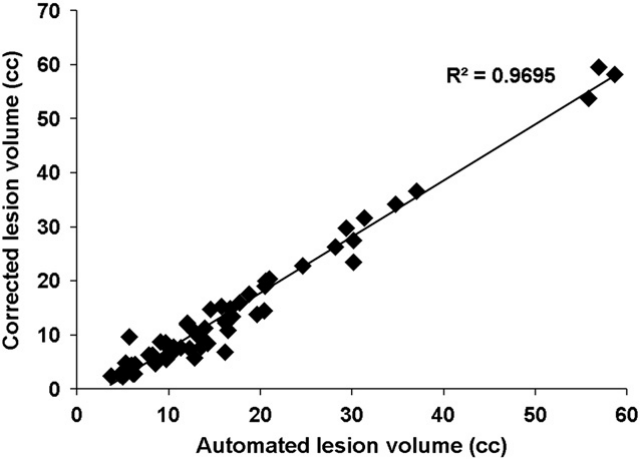
Plot of the estimated lesion volume obtained with the proposed automated technique against the volume corrected by the expert.

Fig. 8 shows the Bland–Altman plot. The mean and mean ± 1.96 ∗ SD are shown in this figure to visually assess the agreement between the automated segmentation and the expert. As can be seen from this figure, the differences in the lesion volumes in most of the subjects lie well within the 95% confidence interval of the mean difference demonstrating good agreement between the two measures. Additionally, the bias below zero (~ 2 cm^3^) observed in this figure indicates the consistent overestimation of lesion volume with the automated technique. Finally, the segmented and corrected volumes were found to be highly correlated as assessed by the Pearson correlation coefficient, R, of 0.98 (Fig. 9).

### 3.3 Validation

**Fig. 10:**
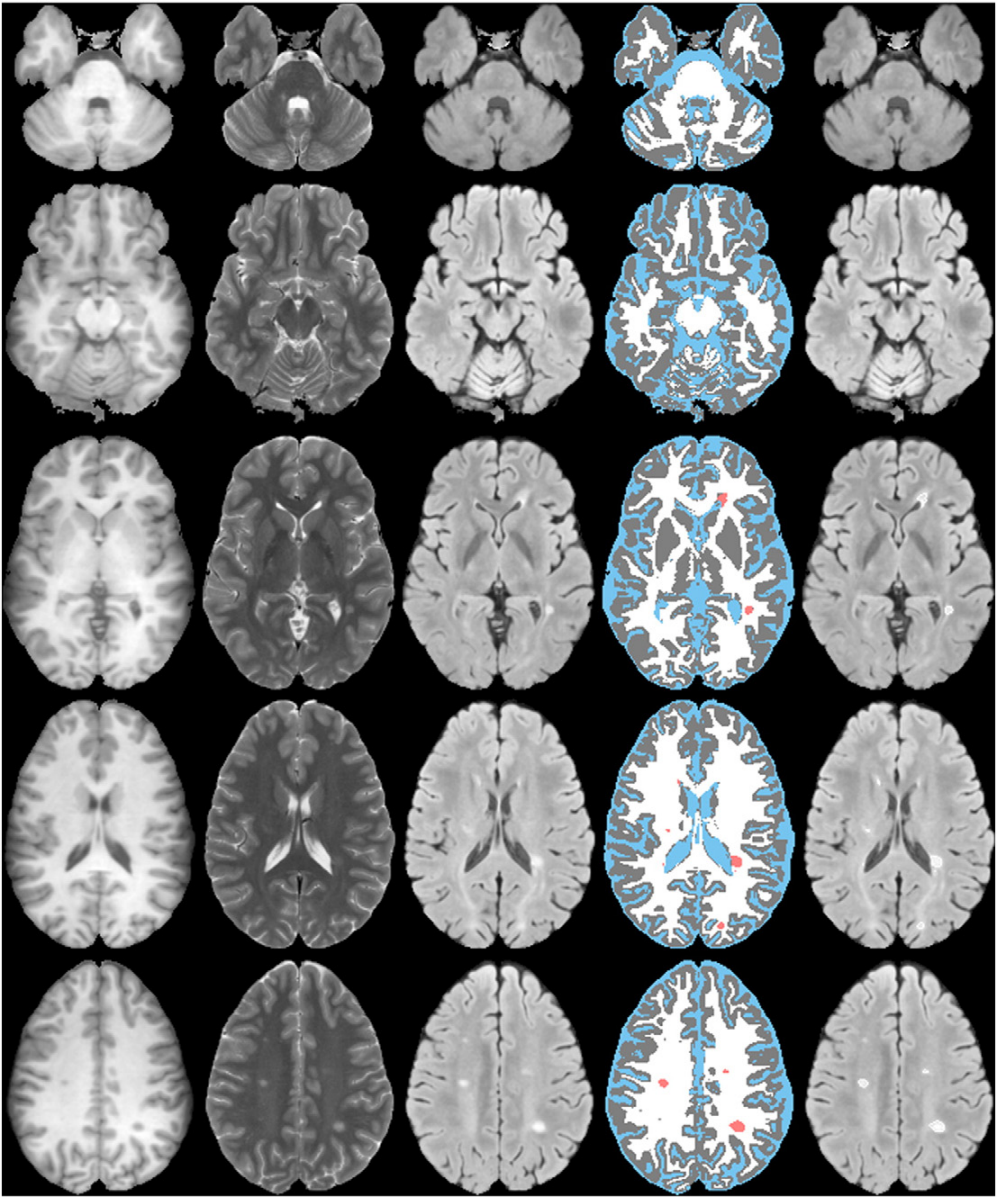
Automated segmentation at various cross-sections of the MICCAI private data CHB_test1_Case13. First column: T1; second column: T2; third column: FLAIR; fourth column: segmented; and fifth column: boundaries of the segmented lesions superimposed on FLAIR images. Color scheme is same as Fig. 2.

**Fig. 11:**
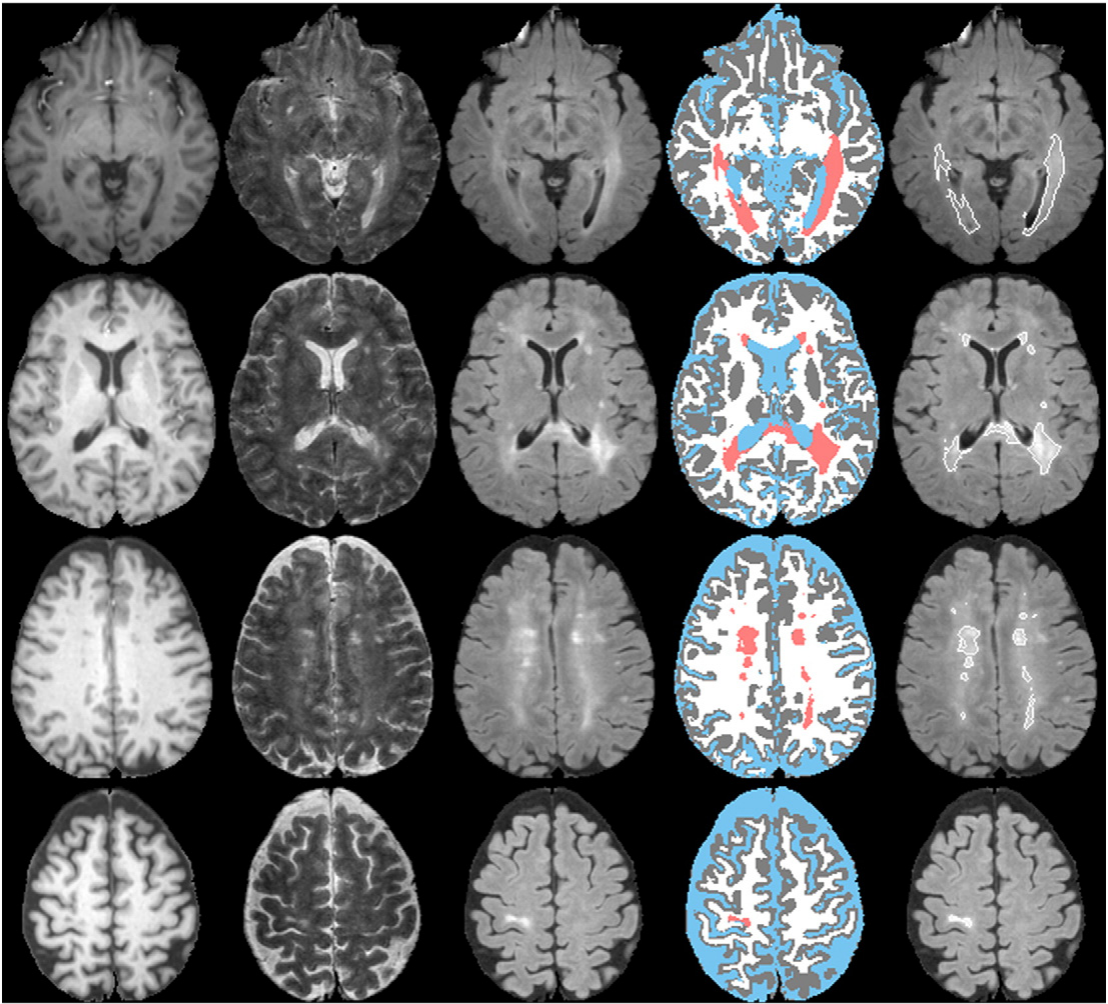
Automated segmentation at various cross-sections of the MICCAI private data UNC_test1_Case12. First column: T1; second column: T2; third column: FLAIR; fourth column: segmented; and fifth column: boundaries of the segmented lesions superimposed on FLAIR images. Color scheme is same as Fig. 2.

MICCAI validation for all the groups is based on the private dataset on the 23 subjects (http://www.ia.unc.edu/MSseg/results_table.php). However, for reasons not clear to us, our data was evaluated on 30 scans. Based on the 30 scans, our technique (team UT-Radiology) received an average score of 78.7635. As an example, Fig. 10 shows the classification of GM, WM, CSF, and THWLs on the CHB_test1_Case13. Note the superior classification of tissues and lesions on this scan. The score sheet provided by MICCAI indicates that our technique's performance was suboptimal on the scans labeled as UNC_test1_Case12, UNC_test1_Case13, and CHB_test1_Case18. Note that these scans are not part of the 23 scans used for evaluating other groups' techniques. As an example, the classification on UNC_test1_Case12 is demonstrated in Fig. 11. Since the ground truth on the MICCAI dataset is not available to us, it is difficult to assess whether the lesion load was underestimated or overestimated by our method compared to the expert lesion segmentation. For fair comparison of the score sheets with other groups, we calculated the average score for our technique using the 23 scans that were used for evaluating the performance of other sites. This improved the score to 82.1739.

## 4 Discussion

We developed and implemented a comprehensive and automated technique for segmenting 3D brain images in MS. As indicated by both qualitative and quantitative results, the proposed technique appears to be accurate and robust. Although the proposed segmentation technique for classifying tissues/lesions is built on our previous technique that was developed for 2D images (Sajja et al., 2006), there are major differences between these two methods. In contrast to the previous method in which the ratio of PD and T2 was used for minimizing the false classifications, in the current method we used the ICBM template to minimize the false lesion classifications. In our previous study, GM and WM were obtained by the application of EM-HMRF algorithm, a parametric classifier on the PD and T2 images, whereas in the current method the same technique was applied to the T1 and T2 images to classify GM, WM, and CSF. In the present study, the classification of the dGM structures was further improved using the ICBM template. In addition, the brain extraction in the current study is fully automatic, whereas the technique proposed by Sajja et al. (2006) relied on a semi-automated technique.

The commonly used brain extraction techniques such as Brain Surface Extractor (BSE) and BET mainly work on the T1 images retaining only the ventricular CSF (Shattuck et al., 2001; Smith, 2002). In contrast our technique works on the T2 images and retains extracortical CSF along with ventricular CSF (Datta and Narayana, 2011; Ramirez et al., 2011) that provides better estimation of total brain volume. Further, GM, WM, and CSF were segmented using the T1 and T2 images with the application of EM-HMRF algorithm.

In general, GM and WM segmentation of the cerebellum is difficult (Datta et al., 2011a). Therefore, it is not uncommon to remove the cerebellum from the images prior to tissue classification (Nakamura et al., 2011; Ramirez et al., 2011). In contrast, our technique classifies cerebellar tissues separately and combines with the remaining part of the brain, thus improving the tissue classification (Datta et al., 2009, 2011a). In the present study, the cerebellum was isolated by deforming the ICBM template with the subject T1 image by ignoring lesions. Perhaps a better strategy would be to co-align the template and subject T1 images by in-painting the THWLs as proposed by Sdika and Pelletier (2009). We intend to implement in-painting of the lesions prior to deforming the ICBM template in our future studies.

Although, the decile based piece-wise linear transformation was applied to standardize the image intensities prior to tissue classification (Nyul et al., 2000), it often overestimates lesions. Our study indicates overestimation of the lesion volume for low lesion load. This trend may be attributed to the intensity standardization which assumes that tissue/lesion intensity on a standard scale is the same across all subjects. This assumption may not be necessarily valid for the MS lesions. However, this intensity standardization is one of the best techniques with least computational time for intensity-based classification (Shah et al., 2011).

Once lesions are classified following the intensity standardization, minimization of false classifications plays a critical role as can be appreciated from Fig. 7 and Tables 2–4. Most of the false lesion classifications occur when the hyperintense GM voxels and the choroid plexus are classified as lesions. There is a very small fraction of false classifications that could result from misalignment of FLAIR with T2 images following the rigid body registration. A deformed template was used to minimize most of these false classifications by mapping the CGM and LVC masks with subject segmented image. These minimization steps dramatically reduced the misclassifications and improved the quantitative metrics.

Many segmentation techniques fail to properly classify the dGM structures (Derakhshan et al., 2010) in MS. A reason for this failure is that these structures appear hypointense on the T2 image (Bakshi et al., 2002). However, as demonstrated in this study, application of the deformation field obtained by registering these images to a template greatly overcame this problem. To summarize, the deformed template was utilized for isolating the cerebellum to obtain better GM and WM classification, minimize false classifications of lesions, and re-classify the dGM structures into GM.

The ideal evaluation of the estimated lesions should be based on multi-raters using techniques such as the simultaneous truth and performance level estimation (STAPLE) (Commowick and Warfield, 2010; Garcia-Lorenzo et al., 2011). In the present study, given the dimensionality of the images, number of subjects, and the amount of time spent by the expert on a single subject, we limited the lesion validation to a single expert. However, we intend to implement the STAPLE technique in our future study.

We validated our technique using the private data available as part of the MS lesion segmentation grand challenge (MICCAI 2008). An average score of 78.7635 on 30 scans was reported for our technique. However, our score increased to 82.1739 when calculated for only 23 scans that were used in evaluating others' techniques. As shown in Fig. 11, the classification of lesions remains suboptimal as the diffuse regions surrounding the focal lesions were not well classified, but should have been classified as part of the lesions. To the best of our knowledge, apart from our technique, the only other reported technique for classifying MS brain into GM, WM, CSF, and lesions was the Topology preserving Anatomical Segmentation (lesion-TOADS) (Shiee et al., 2010). It is worth noting that their lesion classification scored 79.8975 on the 23 private MICCAI 2008 data.

Future studies also include the classification of black holes and Gd-enhanced lesions which are not included in the present study. This could be achieved by applying the morphological grayscale reconstruction techniques proposed by Datta et al. (2007, 2006) and utilizing the segmented THWLs obtained with the technique proposed here. Ignoring the cortical lesions is a limitation of this study. However, cortical lesions are difficult to identify on the FLAIR and T2 images. Double Inversion Recovery (DIR) images are superior in visualizing the cortical lesions (Nelson et al., 2007). Our future studies include the classification of cortical lesions using the procedure described by Datta et al. (2010).

The proposed segmentation technique was implemented on images acquired on a single scanner and true validation of any technique requires multi-center studies. Although we have validated the technique on the MICCAI 2008 data, the scanner details on which the data were acquired are not available to us. Also, this data includes scans from two centers and may not represent the complete heterogeneity related to scanner make and field strength. Our future plans include a multi-center validation study.

All the modules for image pre-processing, segmentation, and non-linear registration of the ICBM template with the subject T1 image were written in IDL (Interactive Data Language) and C programming language. Parameters used for the EM-HMRF and fuzzy connectivity techniques were set using a subset of 60 scans, details of which are described in Sajja et al. (2006) and Datta et al. (2006) The same set of parameters was consistently employed to segment 60 scans from our scanner and MICCAI data. It took approximately 2 h for selecting the training points and generating feature map. Currently, the total computational time for complete segmentation, including the preprocessing and false lesion minimization is 5 h. The computational time can be reduced significantly with the use of parallel and/or GPU (Graphical Processing Unit)-based computation(s).

## 5 Conclusions

We have presented and implemented a comprehensive and fully automated technique for classifying 3D MR brain images in MS. The proposed automated segmentation technique was assessed quantitatively for THWL classification with the use of quantitative metrics, Bland–Altman and regression analyses. The proposed technique should allow accurate estimation of the GM and WM atrophy that is shown to correlate with clinical disability in MS. Since the proposed technique is completely automated, it is expected to be particularly useful in processing a large amount of image of the data that is typically encountered in multi-center clinical trials.

## Figures and Tables

**Table 1:** Sequence parameters, acquisition times, sensitivity encoding (SENSE) factors for isotropic 3D MPRAGE based T1, and turbo-spin echo T2 and FLAIR sequences acquired on Philips 3 T scanner. Here, TE, TR, TI, and ETL represent echo time, repetition time, inversion recovery time, and echo train length. RL and AP stand for the right–left and anterior–posterior directions along which the SENSE factors were applied.

Sequence	TR (ms)	TE (ms)	TI (ms)	ETL	Acquisition time	SENSE
T1	8.1	3.7	–	256	5 min 56 s	2 (RL)
T2	2500	362.9	–	120	5 min 57 s	2 (RL), 2 (AP)
FLAIR	8000	336.8	2400	110	8 min 0 s	2 (RL), 2.5 (AP)

**Table 2:** Quantitative metrics between the corrected lesion volumes and those estimated with the application of the Parzen classification.

Group	vol diff	avg dist (mm)	TPR	FPR
A (31)	184.68 ± 126.37	6.06 ± 2.73	90.42 ± 11.15	92.78 ± 3.65
B (29)	39.37 ± 52.79	2.21 ± 2.00	87.89 ± 9.31	86.83 ± 8.28
Combined (A + B)	114.45 ± 121.68	4.20 ± 3.07	89.20 ± 10.29	89.90 ± 6.95

**Table 3:** Quantitative metrics between the corrected lesion volumes and those estimated following the minimization of false classifications.

Group	vol diff	avg dist (mm)	TPR	FPR
A (31)	56.36 ± 34.55	3.00 ± 1.53	84.41 ± 14.01	37.53 ± 16.37
B (29)	11.79 ± 14.76	0.96 ± 0.60	81.41 ± 11.45	32.58 ± 16.21
Combined (A + B)	34.82 ± 34.86	2.02 ± 1.56	82.96 ± 12.82	35.14 ± 16.34

**Table 4:** Quantitative metrics between the corrected lesion volumes and those estimated following the application of fuzzy connectivity algorithm.

Group	vol diff	avg dist (mm)	TPR	FPR
A (31)	58.19 ± 34.19	2.48 ± 1.26	85.69 ± 14.14	36.83 ± 16.67
B (29)	12.66 ± 14.30	0.75 ± 0.52	83.74 ± 11.10	31.19 ± 14.99
Combined (A + B)	36.18 ± 34.90	1.64 ± 1.30	84.75 ± 12.69	34.10 ± 16.00
