# Supplementary material for: A comprehensive approach to the segmentation of multichannel three-dimensional MR brain images in multiple sclerosis
Source: Neuroimage Clin. 2013 Jan 11;2:184–96. doi: 10.1016/j.nicl.2012.12.007 (PMC3777770; doi:10.1016/j.nicl.2012.12.007)
Supplement: Supplementary file 1 — Supplementary material [file mmc1.pdf]

Ground Truth	UNC Rater								CHB Rater								
All Dataset	Volume Diff.		Avg. Dist.		True Pos.		False Pos.		Volume Diff.		Avg. Dist.		True Pos.		False Pos.		Total
	[%]	Score	[mm]	Score	[%]	Score	[%]	Score	[%]	Score	[mm]	Score	[%]	Score	[%]	Score	
UNC test1 Case01	68.3	90	4.8	90	37.2	73	53.8	77	146.1	79	6.5	87	37.5	73	46.2	82	81
UNC test1 Case03	44.7	93	3.0	94	21.1	63	24.0	95	28.5	96	2.4	95	25.0	66	18.0	99	88
UNC test1 Case04	56.5	92	2.6	95	55.3	83	57.4	75	142.9	79	3.6	93	70.4	91	63.8	71	85
UNC test1 Case05	21.2	97	4.1	92	59.5	85	41.2	85	173.8	75	5.0	90	82.6	98	55.9	76	87
UNC test1 Case06	26.6	96	3.1	94	58.6	85	46.4	81	227.7	67	17.0	65	75.0	94	78.6	62	80
UNC test1 Case07	13.9	98	2.6	95	47.5	78	31.0	91	164.3	76	6.2	87	73.3	93	50.0	79	87
UNC test1 Case08	6.4	99	2.9	94	51.1	80	40.5	85	73.8	89	9.3	81	83.3	99	59.5	73	88
UNC test1 Case09	48.0	93	48.5	0	0.0	51	100.0	49	108.8	84	54.9	0	0.0	51	100.0	49	47
UNC test1 Case10	259.1	62	14.7	70	35.0	71	85.7	57	1198.2	0	24.6	49	50.0	80	92.9	53	55
UNC test1 Case11	16.0	98	1.6	97	35.5	72	8.9	100	27.4	96	0.9	98	44.5	77	5.6	100	92
UNC test1 Case12	10988.1	0	30.2	38	16.7	61	97.5	50	29566.2	0	35.4	27	33.3	70	97.5	50	37
UNC test1 Case13	781.0	0	29.4	39	40.0	74	92.6	53	818.7	0	24.7	49	66.7	89	88.9	56	45
UNC test1 Case14	160.2	77	8.0	84	77.8	96	72.7	65	173.1	75	14.5	70	75.0	94	86.4	57	77
CHB test1 Case01	26.6	96	4.6	91	32.0	70	30.4	91	4.8	99	3.6	92	67.7	90	39.1	86	89
CHB test1 Case02	46.4	93	5.1	90	54.5	82	55.2	76	37.6	94	2.0	96	52.6	81	24.1	95	88
CHB test1 Case03	18.6	97	8.1	83	64.3	88	75.7	64	42.7	94	10.5	78	40.0	74	81.1	60	80
CHB test1 Case04	17.7	97	4.7	90	72.7	93	57.1	75	60.4	91	4.1	92	61.1	86	4.8	100	91
CHB test1 Case05	59.0	91	14.4	70	14.8	60	78.9	62	92.2	86	10.7	78	26.1	66	15.8	100	77
CHB test1 Case06	19.9	97	2.0	96	36.1	72	65.9	70	16.3	98	2.2	95	31.8	70	79.3	61	82
CHB test1 Case07	65.2	90	5.6	88	30.0	69	52.8	77	78.9	88	4.4	91	28.9	68	8.3	100	84
CHB test1 Case08	37.9	94	2.2	95	63.0	87	10.5	100	58.4	91	2.9	94	41.2	75	10.5	100	92
CHB test1 Case09	78.5	89	3.1	94	43.0	76	41.5	84	50.5	93	2.4	95	35.2	71	44.6	82	85
CHB test1 Case10	96.5	86	6.6	86	57.9	84	86.5	57	3.9	99	4.0	92	48.3	79	74.3	64	81
CHB test1 Case11	53.6	92	4.9	90	22.7	64	38.1	86	85.0	88	6.0	88	20.7	63	23.8	95	83
CHB test1 Case12	54.6	92	3.3	93	20.5	63	67.2	69	54.9	92	2.9	94	25.6	66	65.6	70	80
CHB test1 Case13	21.9	97	6.4	87	60.0	86	66.7	69	25.3	96	2.3	95	66.7	89	23.8	95	89
CHB test1 Case15	13.0	98	2.4	95	39.7	74	15.8	100	49.0	93	1.4	97	53.2	82	28.9	92	91
CHB test1 Case16	95.3	86	4.0	92	42.5	76	53.1	77	67.9	90	2.2	95	66.7	89	50.0	79	86
CHB test1 Case17	16.3	98	6.6	86	24.5	65	44.2	83	52.7	92	3.4	93	19.0	62	14.0	100	85
CHB test1 Case18	90.6	87	45.1	7	0.0	51	100.0	49	69.4	90	45.7	6	0.0	51	100.0	49	49
All Average	443.4	86	9.5	80	40.5	74	56.4	75	1123.3	80	10.5	79	46.7	78	51.0	78	79
All UNC	960.8	76	12.0	75	41.2	75	57.8	74	2526.9	63	15.8	69	55.1	83	64.9	70	73
All CHB	47.7	93	7.6	84	39.9	74	55.3	76	50.0	93	6.5	87	40.3	74	40.5	84	83
